# Quantitative CT parameters combined with preoperative systemic inflammatory markers for differentiating risk subgroups of thymic epithelial tumors

**DOI:** 10.1186/s12885-023-11332-0

**Published:** 2023-11-27

**Authors:** Rongji Gao, Jian Zhou, Juan Zhang, Jianzhong Zhu, Tiantian Wang, Chengxin Yan

**Affiliations:** 1 Department of Radiology, The Second Affiliated Hospital of Shandong First Medical University, No.366, Taishan Street, Taian, Shandong Province 271000 China; 2 Department of Radiology, Taian City Central Hospital, No.29, Longtan Road, Taian, Shandong Province 271000 China

**Keywords:** Thymic epithelial tumor, Computed tomography, Systemic inflammatory markers

## Abstract

**Background:**

Thymic epithelial tumors (TETs) are the most common primary neoplasms of the anterior mediastinum. Different risk subgroups of TETs have different prognosis and therapeutic strategies, therefore, preoperative identification of different risk subgroups is of high clinical significance. This study aims to explore the diagnostic efficiency of quantitative computed tomography (CT) parameters combined with preoperative systemic inflammatory markers in differentiating low-risk thymic epithelial tumors (LTETs) from high-risk thymic epithelial tumors (HTETs).

**Methods:**

74 Asian patients with TETs confirmed by biopsy or postoperative pathology between January 2013 and October 2022 were collected retrospectively and divided into two risk subgroups: LTET group (type A, AB and B1 thymomas) and HTET group (type B2, B3 thymomas and thymic carcinoma). Statistical analysis were performed between the two groups in terms of quantitative CT parameters and preoperative systemic inflammatory markers. Multivariate logistic regression analysis was used to determine the independent predictors of risk subgroups of TETs. The area under curve (AUC) and optimal cut-off values were calculated by receiver operating characteristic (ROC) curves.

**Results:**

47 TETs were in LTET group, while 27 TETs were in HTET group. In addition to tumor size and CT value of the tumor on plain scan, there were statistical significance comparing in CT value of the tumor on arterial phase (CTv-AP) and venous phase (CTv-VP), and maximum enhanced CT value (CE_max_) of the tumor between the two groups (for all, *P* < 0.05). For systemic inflammatory markers, HTET group was significantly higher than LTET group (for all, *P* < 0.05), including platelet-to-lymphocyte ratio (PLR), neutrophil-to-lymphocyte ratio (NLR) and systemic immune-inflammation index (SII). Multivariate logistic regression analysis showed that NLR (odds ratio [OR] = 2.511, 95% confidence interval [CI]: 1.322–4.772, *P* = 0.005), CTv-AP (OR = 0.939, 95%CI: 0.888–0.994, *P* = 0.031) and CTv-VP (OR = 0.923, 95%CI: 0.871–0.979, *P* = 0.008) were the independent predictors of risk subgroups of TETs. The AUC value of 0.887 for the combined model was significantly higher than NLR (0.698), CTv-AP (0.800) or CTv-VP (0.811) alone. The optimal cut-off values for NLR, CTv-AP and CTv-VP were 2.523, 63.44 Hounsfeld Unit (HU) and 88.29HU, respectively.

**Conclusions:**

Quantitative CT parameters and preoperative systemic inflammatory markers can differentiate LTETs from HTETs, and the combined model has the potential to improve diagnostic efficiency and to help the patient management.

## Background

Thymic epithelial tumors (TETs), originating from thymic epithelial cells, are the most common primary neoplasms of the anterior mediastinum [1, 2], accounting for approximately 47% of cases [3]. According to the World Health Organization (WHO) classification, TETs are classified into six subtypes: A, AB, B1, B2, B3 and thymic carcinoma (TC), which reflect the oncologic behavior and prognostic features of TETs based on the morphology of epithelial cells and the ratio of epithelial cells to lymphocytes [4–8]. Jeong et al. [9] classified TETs into three subgroups: low-risk thymomas (type A, AB and B1), high-risk thymomas (type B2 and B3) and TC according to the invasiveness and recurrence of the tumor. In addition, some studies performed a simplified classification, defining low-risk thymomas as low-risk TETs (LTETs) and high-risk thymomas and TC as high-risk TETs (HTETs) [6, 10]. Previous studies indicated that there are different therapeutic strategies for different risk subgroups of TETs [11, 12]. For patients with high-risk thymomas and TC, postoperative adjuvant chemoradiotherapy is necessary [13, 14], which can improve survival rates [14–16]. Therefore, accurate and non-invasive preoperative identification of different risk subgroups is of high clinical significance.

Chest computed tomography (CT) examinations are the first choice of an imaging method for clinically suspected thymic lesions, thanks to their low-cost and wide availability [17, 18], providing both qualitative and quantitative parameters. Although several studies have shown that CT signs (tumor morphology, necrosis or cystic degeneration, adjacent tissue infiltration and lymphadenopathy) and quantitative parameters (tumor size, CT attenuation, etc.) can help to distinguish different pathological subtypes of TETs [13, 19, 20], these CT features can not effectively identify WHO classification with satisfactory sensitivity and specificity.

Systemic inflammatory response plays an important role in different stages of tumor development, including initiation, promotion, malignant conversion, invasion and metastasis [21–23]. Tumors stimulate inflammatory cells to release cytokines, which are critical factors in regulating the tumor microenvironment [21, 24, 25]. Systemic inflammatory markers, such as peripheral blood platelet-to-lymphocyte ratio (PLR), neutrophil-to-lymphocyte ratio (NLR) and systemic immune-inflammation index (SII), can reflect the inflammatory and immune status of patients with various tumors. In addition, recent studies have shown that these systemic inflammatory markers can identify different degrees and stages as well as predict the prognosis of many malignant tumors, such as gastric cancer, hepatocellular carcinoma and esophageal squamous cell carcinoma [26–28]. However, no studies have explored whether the preoperative systemic inflammatory markers can be used to differentiate LTETs from HTETs.

Therefore, this study aims to evaluate, for the first time, three new variables of systemic inflammatory markers, including PLR, NLR and SII, combined with quantitative CT parameters to determine their diagnostic efficacy in differentiating LTETs from HTETs.

## Materials and methods

### Patients

**Fig. 1:**
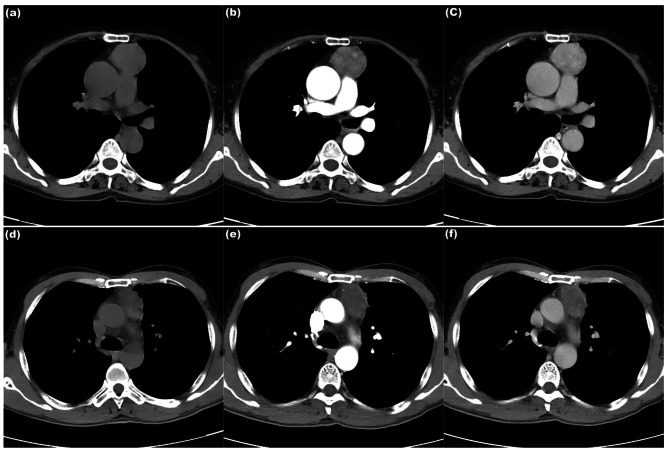
CT features of TETs on axial CT images of mediastinal window. (**a**)-(**c**) A 67-year-old female with type AB thymoma. Axial CT images show a round tumor with well-defined in anterior mediastinum. The CT values of the tumor on unenhanced scan (**a**), arterial phase (**b**) and venous phase (**c**) are 44.07 Hounsfeld Unit (HU), 95.15HU and 125.36HU, respectively, while the maximum enhanced CT value (CE_max_) of the tumor is 81.29HU. (**d**)-(**f**) A 50-year-old male with type B3 thymoma. Axial CT images show an oval tumor with ill-defined in anterior mediastinum. The CT values of the tumor on unenhanced scan (**d**), arterial phase (**e**) and venous phase (**f**) are 40.76HU, 45.62HU and 63.03HU, respectively, while the CE_max_ of the tumor is 22.27HU

**Fig. 2:**
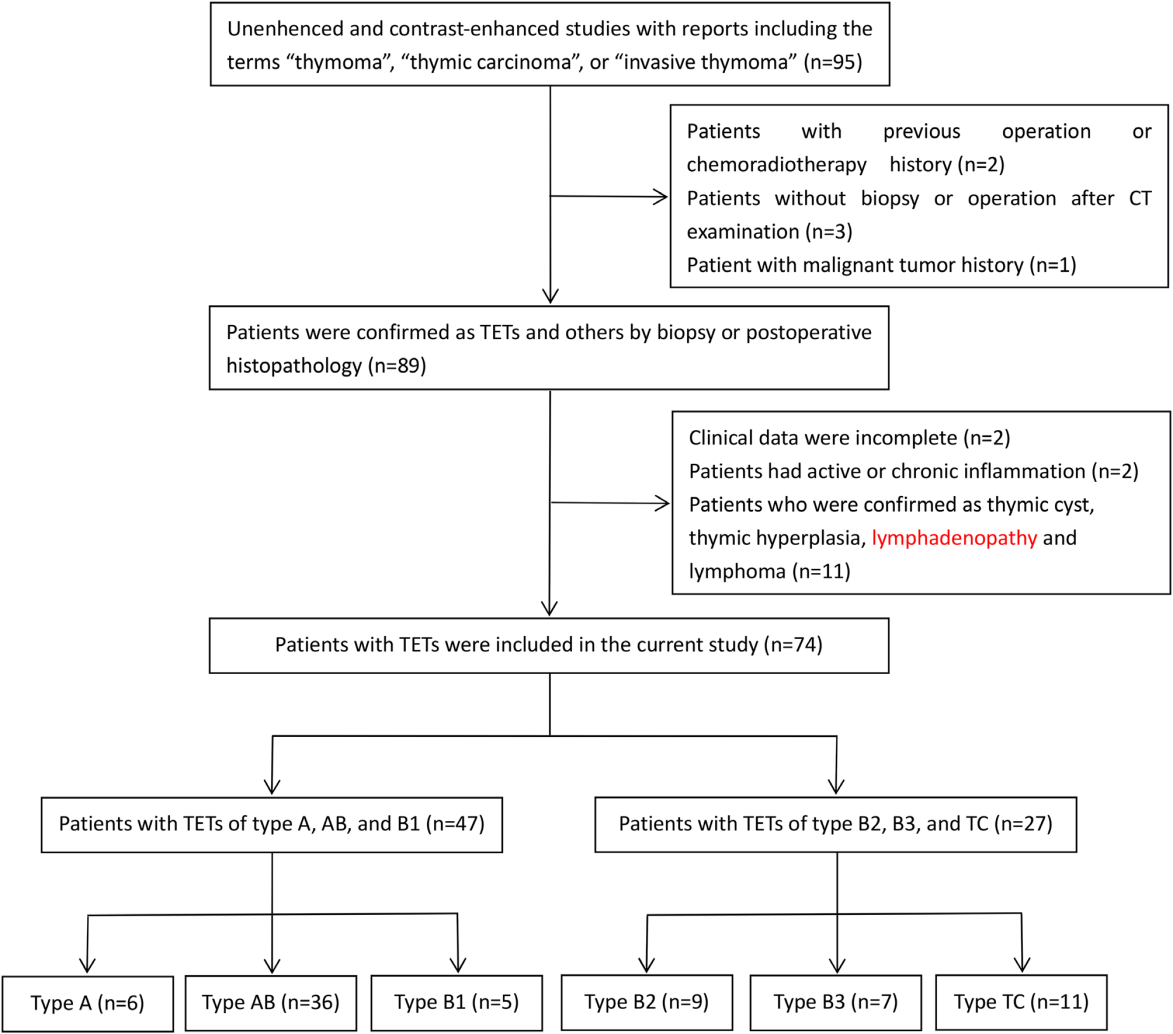
The flowchart of case collection. Numbers in parentheses are the number of patients

We retrospectively analyzed our clinical database of patients with TETs who underwent biopsy or operation at the Second Affiliated Hospital of Shandong First Medical University from January 2013 to October 2022. This study was approved by the Institutional Ethics Committee of the Second Affiliated Hospital of Shandong First Medical University (No. 2021-086) and informed consent was obtained from all patients. The inclusion criteria are as follows: (1) biopsy or operation should be performed within 1–2 weeks after chest unenhanced and arteriovenous dual contrast-enhanced CT scan, (2) routine laboratory blood test should be performed within 1 week before biopsy or operation, (3) postoperative histopathology is confirmed as TETs, and (4) there is no any previous operation or chemoradiotherapy history. The exclusion criteria are as follows: (1) incomplete clinical data, (2) active infection or chronic inflammatory disease and (3) malignant tumor history. In this study, we used the simplified classification, classifying low-risk thymomas (type A, AB, and B1) as low-risk TETs (LTETs) and high-risk thymomas (type B2 and B3) and TC as high-risk TETs (HTETs) (Fig. 1). The flowchart of case collection is shown in Fig. 2.

### CT examinations

A 256-slice CT scanner (Brilliance iCT, Philips Healthcare, Cleveland, USA) and a 128- slice CT scanner (LightSpeed VCT, GE Healthcare, Asahigaoka, Japan) were used to perform plain and contrast-enhanced CT scans with the following parameters: 120 KVp tube voltage, 250 mAs effective dose, 0.993 pitch, 512 × 512 matrix, 363 mm display field of view, and 120 KVp tube voltage, 90–350 mA, 0.984 pitch, 512×512 matrix, 350 mm display field of view, respectively. All CT examinations were performed from the apex to the base of the lung. For the contrast-enhanced CT scan, all patients were injected with 350 mg/ml of iodine contrast agent (Ioversol, Jiangsu Hengrui Pharmaceutical, Jiangsu, China) at a dose of 1.0 ml/kg body weight and a flow rate of 3.5ml/s by the high-pressure injector (CT motion-XD 8000, Irich Medical, Ulm, Germany). The venous phase (VP) scan started about 40 s after the arterial phase (AP) with automatic scanning by contrast agent tracing technique.

### Imaging analysis

The Digital Imaging and Communications in Medicine (DICOM) format CT images were transferred to the workstation IntelliSpace Portal (version 5.0.2.40009, Philips Healthcare, The Netherlands) for mediastinal window setting (window width, 350 Hounsfeld Unit [HU], window level, 40HU). Two senior radiologists (with 10 and 15 years of experience in chest radiology, respectively) interpreted the images and measured the quantitative CT parameters on the plain scan, AP and VP of the monochromatic and material images independently. Neither of the radiologists was unaware of the information about the patients and pathological classification.

The quantitative CT parameters include the following: (1) the maximum diameter (Md) of the tumor, (2) the longest diameter perpendicular to the maximum diameter (Ldp), (3) the CT values of the tumor on plain scan (CTv-C-), (4) the CT values of the tumor on arterial phase (CTv-AP) and (5) the CT values of the tumor on venous phase (CTv-VP). All the measurements of the diameter and CT values were respectively performed three times on the maximum and the continuous axial-sectional images to obtain average values. The CT values of the region of interest (ROI) were measured as the solid component of the tumor, avoiding vascular, necrosis, cystic degeneration, calcification, and artifact caused by high concentration contrast agents in the ascending aorta and aortic pulsation. The maximum enhanced CT value (CE_max_) of the tumor was calculated by the following equation:

1 $${\text{C}}{{\text{E}}_{\max }} = {\text{CT}}{{\text{v}}_{{\text{enhancement}}}} - {\text{CT}}{{\text{v}}_{{\text{unenhancement}}}}$$

where CTv_enhancement_ and CTv_unenhancement_ were the maximum enhanced CT value (CTv-AP or CTv-VP) and CTv-C-, respectively.

### Data collection

Relevant clinical data, including age, gender, platelet count, lymphocyte count and neutrophil count, were collected. All routine laboratory blood tests were done within 1 week before the operation. PLR and NLR were defined as the platelet or neutrophil count divided by lymphocyte count. SII was calculated using the following equation:

2 $${\text{SII = (Plt}} \times {\text{Neu)/Lym}}$$

where Plt, Neu and Lym represented the count of platelet, neutrophil and lymphocyte, respectively.

### Statistical analysis

SPSS Version 20.0 statistical analysis software (IBM, Armonk, New York, USA) and GraphPad Prism Version 9.0.0 software (GraphPad Software Inc., San Diego, California, USA) were used to analyze the results and plot the figures and receiver operating characteristic (ROC) curves. The counting data was expressed as the number of cases, which was tested by chi-square test. Kolmogorov-Smirnov test was used to test the quantitative data, which was in accordance with normal distribution and expressed as mean ± standard deviation, otherwise expressed as median (first quartile, third quartile), and then with the Levene test for variance homogeneity analysis. Two-sample t-test was performed to assess the difference in quantitative data (age, Plt, PLR, Md, Ldp and CTv-C-), whereas the variables of Lym, Neu, NLR, SII, CTv-AP, CTv-VP and CE_max_ were compared by Mann-Whitney U test. Univariate and multivariate logistic regression analysis were used to determine the independent predictors for different risk subgroups of the variables with statistical significance. The regression equation of combined model was established, and the diagnostic efficacy of the regression equation was analyzed by ROC curve. The area under curve (AUC), sensitivity, specificity and optimal cut-off value were calculated. The diagnostic efficacy is considered high if AUC is greater than 0.9, medium if AUC within 0.7–0.9, and low if AUC within 0.5–0.7. *P* < 0.05 is considered statistically significant.

## Results

### Clinical data

**Fig. 3:**
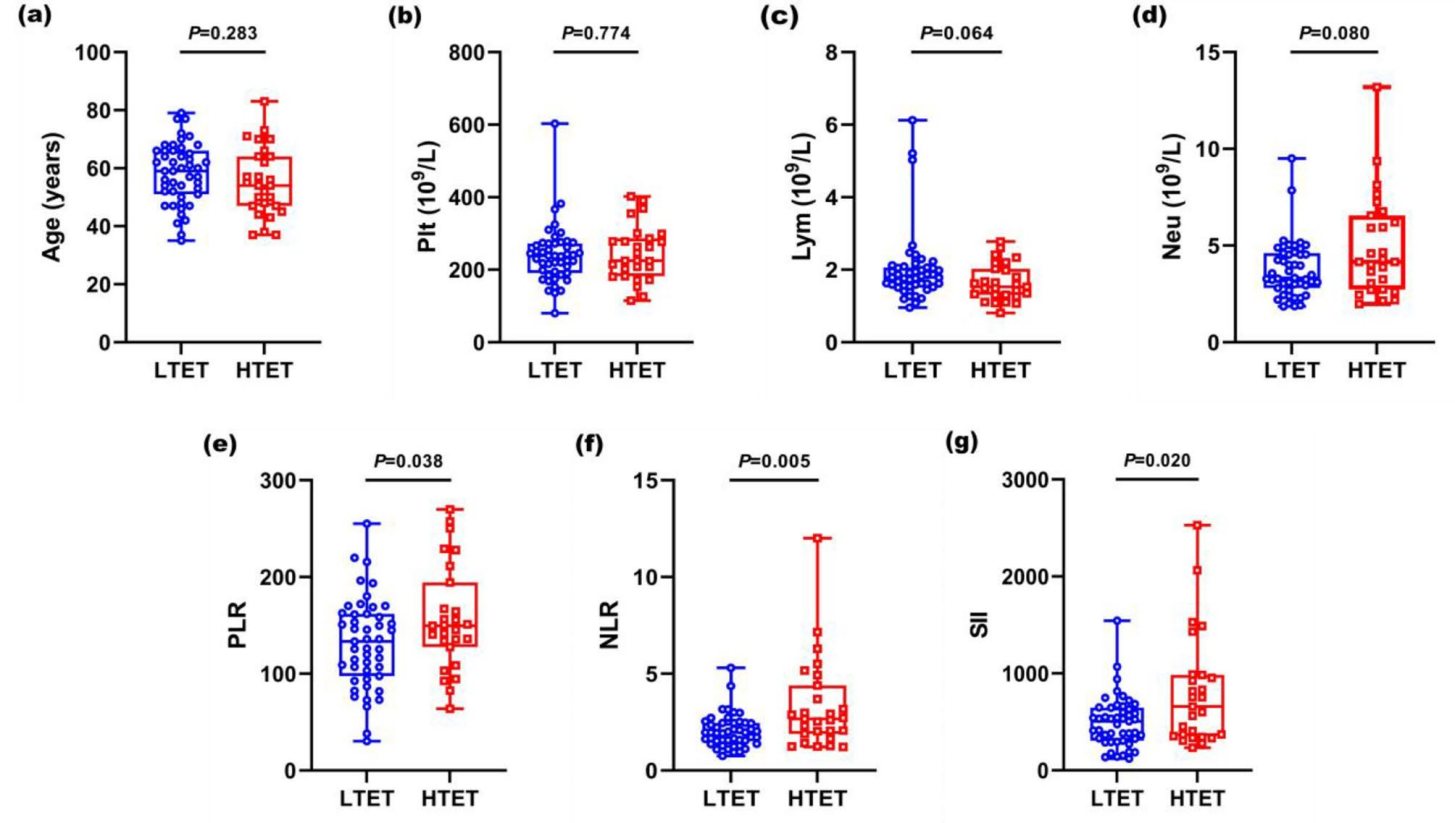
The figures of clinical data for differentiating LTET group from HTET group. (**a**) As for age, the difference is no statistical significance between the two groups (*P* = 0.283). (**b**)-(**d**) In terms of Plt, Lym and Neu, there is no significant difference between the two groups (for all of these, *P* > 0.05). (**e**)-(**g**) With regard to PLR, NLR and SII, HTET group is significantly higher than LTET group (for all of these, *P* < 0.05)

The clinical data of the patients for LTET group and HTET group are shown in Table 1 and plotted in Fig. 3. A total of 74 patients (31 males and 43 females, mean age, 57.16 ± 11.03 years, age range, 35–83 years old) with TETs were included in this study. Among them, 66 patients underwent operation and 8 patients underwent biopsy. According to the WHO classification, the numbers of type A, AB, B1, B2, B3 thymomas and TC were 6, 36, 5, 9, 7 and 11, respectively. There was no significant difference in age between the two groups (*P* = 0.283). However, LTET group appeared more in females (*P* = 0.022). There was no significant difference between the two groups in terms of Plt, Lym and Neu (*P* = 0.774, *P* = 0.064, *P* = 0.080, respectively). However, LTET group was significantly lower than HTET group in terms of PLR, NLR and SII (*P* = 0.038, *P* = 0.005, *P* = 0.020, respectively).

**Table 1:** Clinical data about LTET group and HTET group

	LTET	HTET	χ^2^ / *t* / *Z* value	*P* value
**No. patients**	47	27	-	-
**Gender**			5.267	0.022^a^
Male	15	16		
Female	32	11		
**Age, years**	58.17 ± 11.005	55.84 ± 11.806	1.083	0.283^b^
**Plt, 10** ^**9**^ **/L**	241.40 ± 81.458	241.63 ± 74.343	-0.288	0.774^b^
**Lym, 10** ^**9**^ **/L**	1.770(1.535,2.068)	1.620(1.315,2.035)	-1.752	0.064^c^
**Neu, 10** ^**9**^ **/L**	3.741 ± 1.513	4.779 ± 2.461	-1.752	0.080^c^
**PLR**	134.367 ± 47.843	149.742 ± 53.766	-2.116	0.038^b^
**NLR**	1.951(1.493,2.483)	2.491(1.897,3.566)	-2.824	0.005^c^
**SII**	490.637(321.015,647.673)	582.937(346.323,945.956)	-2.319	0.020^c^

LTET, low-risk thymic epithelial tumor, HTET, high-risk thymic epithelial tumor, Plt, platelet, Lym, lymphocyte, Neu, neutrophil, PLR, platelet-to-lymphocyte ratio, NLR, neutrophil-to-lymphocyte ratio, SII, systemic immune-inflammation index. - no statics and *P* value. ^a^*P* value was calculated by chi-square test. ^b^*P* value was calculated by two-sample t-test. ^c^*P* value was calculated by Mann-Whitney U test

### Quantitative CT parameters

**Fig. 4:**
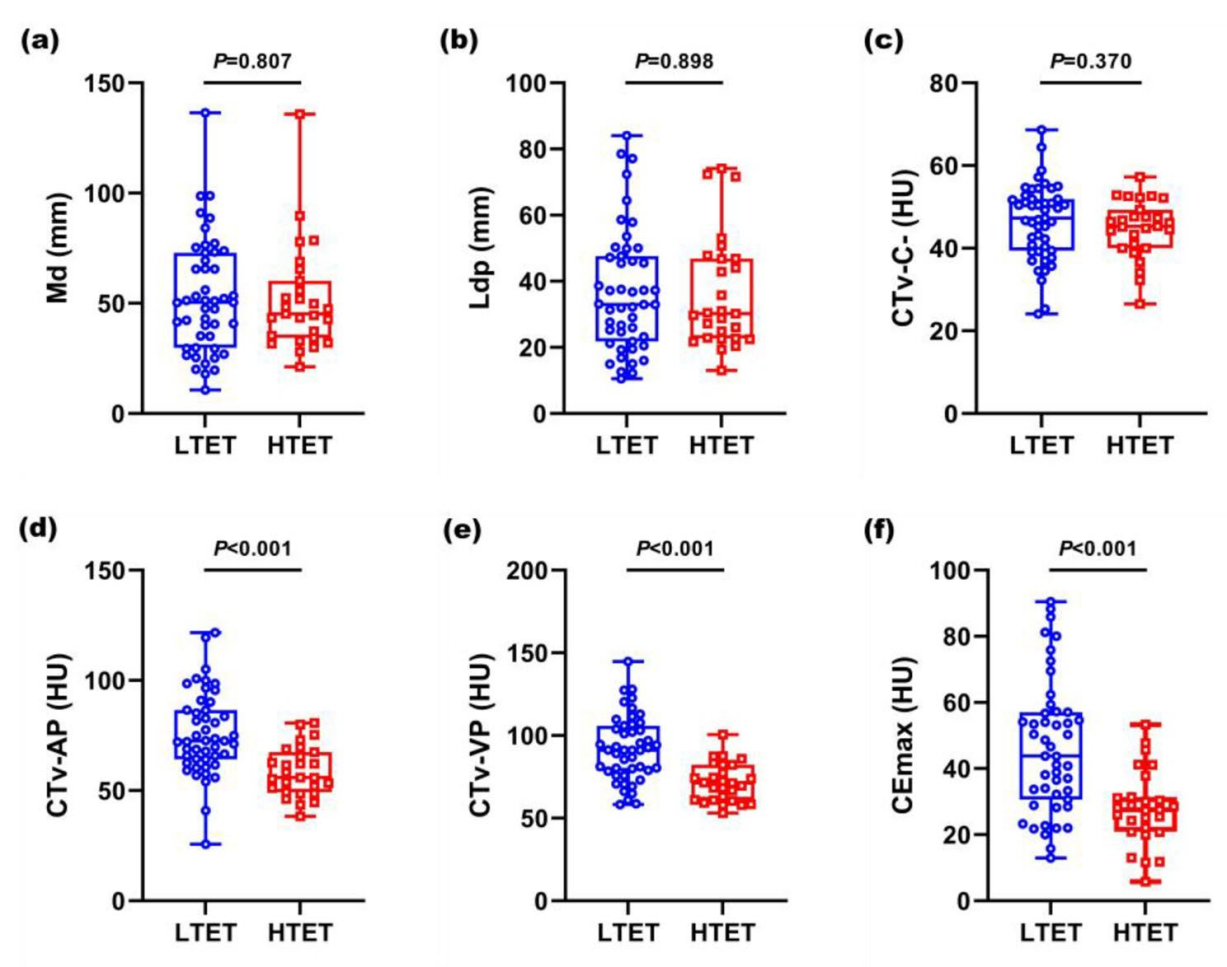
The figures of quantitative CT parameters for differentiating LTET group from HTET group. (**a**)-(**c**) As for the Md, Ldp and CTv-C-, the differences are no statistical significance between the two groups (for all of these, *P* > 0.05). (**d**)-(**f**) The contrast-enhanced CT value of LTET group is significantly higher than that of HTET group (for all of these, *P* < 0.05), including CTv-AP, CTv-VP and CE_max_

A comparison of quantitative CT parameters between LTET group and HTET group is summarized in Table 2 and plotted in Fig. 4. For the CT values of the tumor, including CTv-AP, CTv-VP and CE_max_, LTET group was higher than HTET group with significant difference (for all of these, *P* < 0.001). However, there were no significant difference in the size of the tumor (including Md and Ldp) and CTv-C- (*P* = 0.807, *P* = 0.898, *P* = 0.370, respectively).

**Table 2:** Summary of quantitative CT parameters of LTET group and HTET group

	LTET	HTET	*t* / *Z* value	*P* value
**Md, mm**	55.216 ± 25.352	52.214 ± 24.811	0.245	0.807^b^
**Ldp, mm**	36.177 ± 17.291	37.219 ± 18.817	0.128	0.898^b^
**CTv-C-, HU**	46.240 ± 9.449	45.422 ± 6.975	0.903	0.370^b^
**CTv-AP, HU**	76.069 ± 19.142	60.952 ± 13.017	-4.273	< 0.001^c^
**CTv-VP, HU**	93.780 ± 19.107	72.082 ± 12.971	-4.430	< 0.001^c^
**CE**_**max**_ **, HU**	48.446 ± 20.034	28.152 ± 11.500	-4.014	< 0.001^c^

LTET, low-risk thymic epithelial tumor, HTET, high-risk thymic epithelial tumor, Md, maximum diameter, Ldp, longest diameter perpendicular to maximum diameter, CTv-C-, CT value of the tumor on plain scan, CTv-AP, CT value of the tumor on arterial phase, CTv-VP, CT value of the tumor on venous phase, CE_max_, maximum enhanced CT value. ^b^*P* value was calculated by two-sample t-test. ^c^*P* value was calculated by Mann-Whitney U test

### The univariate and multivariate logistic regression analysis

The results of univariate and multivariate logistic regression analysis between LTET group and HTET group are summarized in Table 3. Univariate logistic regression analysis showed that the variables with significant differences in clinical data and CT quantitative parameters between the two groups, including gender, PLR, NLR, SII, CTv-AP, CT-VP and CE_max_, were identified as risk predictors. After multivariate logistic regression analysis by forward stepwise method, only NLR (X_3_) (odds ratio [OR] = 2.511, 95% confidence interval [CI]: 1.322–4.772, *P* = 0.005), CTv-AP (X_5_) (OR = 0.939, 95%CI: 0.888–0.994, *P* = 0.031) and CTv-VP (X_6_) (OR = 0.923, 95%CI: 0.871–0.979, *P* = 0.008) were identified as independent risk predictors, and the regression equation of combined model was:

Logit(*P)* = 7.530 + 0.921X_3_-0.062X_5_-0.080X_6_

where X_3_, X_5_ and X_6_ represented the NLR, CTv-AP and CTv-VP, respectively.

**Table 3:** Univariate and multivariate logistic regression analysis between LTET group and HTET group

	Univariate analysis			Multivariate analysis	
	OR (95%CI)	*P* value		OR (95%CI)	*P* value
**Gender (X**_**1**_ **)**	3.103(1.162–8.289)	0.024		-	-
**PLR (X**_**2**_ **)**	1.010(1.000-1.021)	0.044		-	-
**NLR (X**_**3**_ **)**	1.945(1.215–3.116)	0.006		2.511(1.322–4.772)	0.005
**SII (X**_**4**_ **)**	1.002(1.000-1.003)	0.010		-	-
**CTv-AP (X**_**5**_ **)**	0.926(0.887–0.967)	0.001		0.939(0.888–0.994)	0.031
**CTv-VP (X**_**6**_ **)**	0.921(0.882–0.961)	< 0.001		0.923(0.871–0.979)	0.008
**CE**_**max**_ **(X**_**7**_ **)**	0.928(0.890–0.968)	< 0.001		-	-

PLR, platelet-to-lymphocyte ratio, NLR, neutrophil-to-lymphocyte ratio, SII, systemic immune-inflammation index, CTv-AP, CT value of the tumor on arterial phase, CTv-VP, CT value of the tumor on venous phase, CE_max_, maximum enhanced CT value, OR, odds ratio, CI, confidence interval. - no statics and *P* value

### ROC curves and cut-off values of the variables

**Fig. 5:**
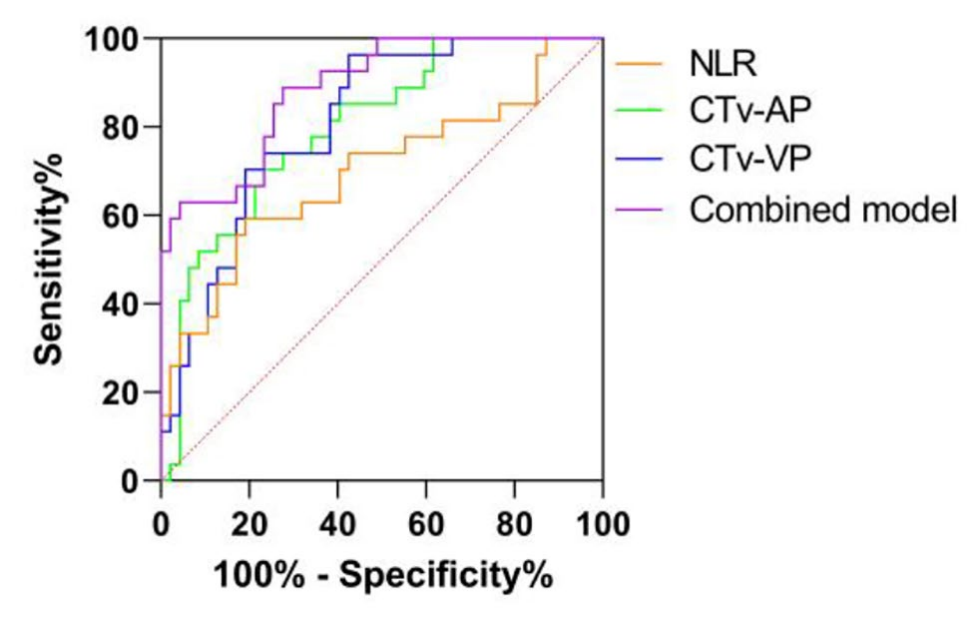
ROC curve for differentiating LTET group from HTET group. The AUC values of combined model compared to NLR, CTv-AP and CTv-VP alone are 0.887 (95%CI, 0.813–0.960), 0.698 (95%CI, 0.567–0.830), 0.800 (95%CI, 0.698–0.902) and 0.811 (95%CI, 0.714–0.907), respectively. The diagnostic efficacy of the combined model is medium, which is 0.7–0.9, and is better than NLR, CTv-AP or CTv-VP alone

The results of ROC curve analysis for LTET group and HTET group are summarized in Table 4 and ROC curves for identifying LTET group from HTET group are plotted in Fig. 5. The AUC value of combined model was 0.887 (95%CI, 0.813–0.960, *P* < 0.001), which was significantly higher than that of NLR (AUC = 0.698, 95%CI: 0.567–0.830, *P* = 0.005), CTv-AP (AUC = 0.800, 95%CI: 0.698–0.902, *P* < 0.001) and CTv-VP (AUC = 0.811, 95%CI: 0.714–0.907, *P* < 0.001). The combined model with medium diagnostic efficacy revealed 88.9% sensitivity and 72.3% specificity. The optimal cut-off values of NLR, CTv-AP, CTv-VP and combined model were 2.523, 63.44HU, 88.29HU and 0.302, respectively.

**Table 4:** ROC curve analysis for differentiating LTET group from HTET group

	AUC	95% CI	*P* value	Sensitivity	Specificity	Youden index	Cut-off value
**NLR**	0.698	0.567–0.830	0.005	0.593	0.809	0.402	2.523
**CTv-AP**	0.800	0.698–0.902	< 0.001	0.704	0.766	0.470	63.44
**CTv-VP**	0.811	0.714–0.907	< 0.001	0.963	0.574	0.537	88.29
**Combined model**	0.887	0.813–0.960	< 0.001	0.889	0.723	0.612	0.302

NLR, neutrophil-to-lymphocyte ratio, CTv-AP, CT value of the tumour on arterial phase, CTv-VP, CT value of the tumour on venous phase, AUC, area under curve, CI, confidence interval

## Discussion

It is well known that the risk degree of TETs is related to the prognosis of patients. Type A and AB thymomas usually show the behavioral characteristics of benign tumors. Type B1 thymoma is a low-grade malignant tumor with a good prognosis as the 10-year survival rate of the patients is more than 90% [13]. Type B2 thymoma is more malignant, while type B3 thymoma and TC are malignant tumors with even a poorer prognoses. Patients with type B2, B3 thymoma and TC generally require adjuvant chemoradiotherapy [10, 13, 19]. Therefore, preoperative non-invasive prediction of different risk subgroups of TETs is extremely important to guide clinical treatment strategies for patients with TETs. In this study, we retrospectively analyzed data of 74 patients with TETs to explore the diagnostic efficacy of the combination of quantitative CT parameters and preoperative systemic inflammatory markers in predicting risk subgroups of TETs.

There are different opinions on differentiating LTETs from HTETs in the demographic characteristics of patients with TETs. In this study, there was no significant age difference between LTETs and HTETs, although LTETs were more frequent and significantly different in females, which is basically consistent with a previous study [29]. However, Hu et al. [13] reported that type B3 thymoma and TC appeared more in male gender (*P* = 0.013), and several studies demonstrated that there was no significant difference between the two groups in gender [10, 30]. The difference in gender between these studies may be due to different geography, environment, TETs subsets or other reasons. Therefore, there is a need for further study on the different demographic characteristics of the patients with TETs in differentiating LTETs from HTETs around the world.

In general, the higher degree of malignancy the tumor, the faster it grows and the larger it becomes. However, our findings are contrary to this view and similar to the results of previous studies [10, 31], indicating that the size of HTET was smaller than that of LTET without significant difference and that tumor size may not be regarded as a reliable biomarker of malignancy. Analysis of the reason may be that comparing to HTET, LTET is less likely to infiltrate adjacent tissues, its clinical symptoms appear relatively late, and the tumor is relatively large at the time of symptoms onset. CT enhancement plays an important role in the diagnosis of TETs since it can better reveal the morphology of the tumor, infiltration of adjacent tissues and distant metastasis, while reflecting the blood supply of the tumor. Hu et al. [13] demonstrated that the CE_max_ of low-risk TETs (type A and AB thymomas) was higher than that of high-risk TETs (type B1, B2, B3 thymomas and TC) with significant difference, and the optimal cut-off value was 25.5HU with 78.8% sensitivity and 68.5% specificity. Despite different groups, our study also confirmed this. Similarly, CTv-AP and CTv-VP of LTETs were significantly higher than that of HTETs. This is basically consistent with a study by Tomiyama et al. [32] which demonstrated that type A and AB thymomas were associated with a higher degree of CT enhancement. Similar to tumor size, this also suggests that a high degree of CT enhancement does not mean a higher degree of malignancy. The difference in the degree of CT enhancement between LTETs and HTETs may be related to the pathological features of the tumor. Pan et al. [33] studied the clinicopathologic and immunohistochemical features of spindle cell thymoma (type A) and mixed spindle/lymphocytic thymoma (type AB) and found that type A often presents hemangiopericytic and microcystic patterns, which may account for the higher degree of CT enhancement in LTETs.

In recent years, systemic inflammatory markers have been widely used not only for benign lesions but also for malignant tumors [34–38]. Systemic inflammatory response may play an important role in the occurrence and development of cancer. Platelet count, lymphocyte count and neutrophil count by routine laboratory blood test may help to understand the systemic inflammatory and immune status of the patients with TETs. However, these inflammatory parameters are susceptible to individual and other factors. Thus, a combination of systemic inflammatory markers such as PLR, NLR and SII may theoretically be more reliable [39]. Tong et al. [40] reported that NLR and SII were the independent factors related to the overall survival (OS) of patients with stage III non-small cell lung cancer and patients with high NLR and SII had significantly worse OS. Li et al. [41] found that PLR and NLR of healthy volunteers were significantly lower than that of patients with laryngeal carcinoma and patients with lower PLR and NLR showed a lower 5-year mortality than those with higher PLR and NLR in terms of survival. Although a study by Wang et al. [42] reported that NLR and monocyte-to-lymphocyte ratio of patients with TETs were significantly higher than that of healthy volunteers, no study to date has investigated the relationship between the preoperative systemic inflammatory markers (including PLR, NLR and SII) and different risk subgroups of TETs. Based on this, we have carried out the preliminary study on this aspect and found that HTETs were significantly higher than LTETs in these systemic inflammatory markers. However, PLR and SII were not independent predictors for identifying LTETs and HTETs by multivariate logistic regression analysis, indicating that NLR is a relevant biomarker of systemic inflammation in different risk subgroups of TETs.

Univariate and multivariate logistic regression analysis showed that only NLR, CTv-AP and CTv-VP were the independent predictors of the risk subgroups of TETs, and ROC analysis indicated that the combined model had better diagnostic efficiency than quantitative parameters of CT enhancement or NLR alone with medium diagnostic efficacy, revealing 88.9% sensitivity and 72.3% specificity.

### Limitations

Firstly, there was a potential selection bias because of the retrospective design of the study. Secondly, CT images were obtained from different manufacturers of CT scanners with different scanning protocols, which might have affected the CT values. Thirdly, the low number limitation of high-risk thymomas and TC might have affected the results. We integrated them into HTET group rather than separating them for statistical analysis due to the relatively insufficient cases.

## Conclusions

The risk subgroups of TETs are associated with CT values of the tumor on contrast-enhanced phase and preoperative systematic inflammatory markers. Combination of quantitative CT parameters and preoperative systemic inflammatory markers can distinguish LTETs from HTETs, and the combined model has the potential to improve diagnostic efficiency and clinical value.

## Data Availability

The datasets used and analyzed during the current study are available from the corresponding author on reasonable request.

## Abbreviations

AP: Arterial phase
AUC: Area under curve
CE_max_: Maximum enhanced CT value
CI: Confidence interval
CT: Computed tomography
CTv-AP: CT value of the tumor on arterial phase
CTv-C-: CT value of the tumor on plain scan
CTv-VP: CT value of the tumor on venous phase
DICOM: Digital Imaging and Communications in Medicine
HTETs: High-risk thymic epithelial tumors
HU: Hounsfeld unit
Ldp: Longest diameter perpendicular to maximum diameter
LTETs: Low-risk thymic epithelial tumors
Lym: Lymphocyte
Md: Maximum diameter
Neu: Neutrophil
NLR: Neutrophil-to-lymphocyte ratio
OR: Odds ratio
OS: Overall survival
PLR: Platelet-to-lymphocyte ratio
Plt: Platelet
ROC: Receiver operating characteristic
ROI: Region of interest
SII: Systemic immune-inflammation index
TC: Thymic cancer
TETs: Thymic epithelial tumors
VP: Venous phase
WHO: World Health Organization

## References

[1] Carter BW, Tomiyama N, Bhora FY, Rosado de Christenson ML, Nakajima J, Boiselle PM, Detterbeck FC, Marom EM (2014). A modern definition of mediastinal compartments. J Thorac Oncol.

[2] Yu C, Li T, Yang X, Zhang R, Xin L, Zhao Z, Cui J (2022). Contrast-enhanced CT-based radiomics model for differentiating risk subgroups of thymic epithelial tumors. BMC Med Imaging.

[3] Engels EA (2010). Epidemiology of thymoma and associated malignancies. J Thorac Oncol.

[4] Marx A, Chan JK, Coindre JM, Detterbeck F, Girard N, Harris NL, Jaffe ES, Kurrer MO, Marom EM, Moreira AL, Mukai K, Orazi A (2015). The 2015 World Health Organization classification of tumors of the Thymus: continuity and changes. J Thorac Oncol.

[5] Roden AC, Yi ES, Jenkins SM, Edwards KK, Donovan JL, Lewis JE, Cassivi SD, Marks RS, Garces YI, Aubry MC (2015). Reproducibility of 3 histologic classifications and 3 staging systems for thymic epithelial neoplasms and its effect on prognosis. AM J Surg Pathol.

[6] Chen G, Marx A, Chen WH, Yong J, Puppe B, Stroebel P, Mueller-Hermelink HK (2002). New WHO histologic classification predicts prognosis of thymic epithelial tumors: a clinicopathologic study of 200 thymoma cases from China. Cancer.

[7] Tamburini N, Maniscalco P, Migliorelli A, Nigim F, Quarantotto F, Maietti E, Cavallesco G (2020). Thymic epithelial tumors: prognostic significance and relationship between histology and the New TNM staging system. Thorac Cardiovasc Surg.

[8] Rioja P, Ruiz R, Galvez-Nino M, Lozano S, Valdiviezo N, Olivera M, Cabero O, Guillen ME, De La Guerra A, Amorin E, Barrionuevo C, Mas L (2021). Epidemiology of thymic epithelial tumors: 22-years experience from a single-institution. Thorac cancer.

[9] Jeong YJ, Lee KS, Kim J, Shim YM, Han J, Kwon OJ (2004). Does CT of thymic epithelial tumors enable us to differentiate histologic subtypes and predict prognosis?. AJR Am J Roentgenol.

[10] Yasaka K, Akai H, Nojima M, Shinozaki-Ushiku A, Fukayama M, Nakajima J, Ohtomo K, Kiryu S (2017). Quantitative computed tomography texture analysis for estimating histological subtypes of thymic epithelial tumors. Eur J Radiol.

[11] Galli G, Trama A, Abate-Daga L, Brambilla M, Garassino MC, Fabbri A (2020). Accuracy of pathologic diagnosis for thymic epithelial tumors: a brief report from an italian reference Center. Lung Cancer.

[12] Yu C, Li T, Yang X, Zhang R, Xin L, Zhao Z, Cui J (2022). Contrast-enhanced CT-based radiomics model for differentiating risk subgroups of thymic epithelial tumors. BMC Med Imaging.

[13] Hu YC, Wu L, Yan LF, Wang W, Wang SM, Chen BY, Li GF, Zhang B, Cui GB (2014). Predicting subtypes of thymic epithelial tumors using CT: new perspective based on a comprehensive analysis of 216 patients. Sci Rep.

[14] Alkaaki A, Abo Al-Saud A, Di Lena É, Ramirez-GarciaLuna JL, Najmeh S, Spicer J, Ferri L, Mulder D, Sirois C, Cools-Lartigue J. Factors predicting recurrence in thymic epithelial neoplasms. Eur J Cardiothorac Surg. 2022;62:ezac274. 10.1093/ejcts/ezac274 35471455

[15] Girard N (2012). Chemotherapy and targeted agents for thymic malignancies. Expert Rev Anticancer Ther.

[16] Girard N, Ruffini E, Marx A, Faivre-Finn C, Peters S (2015). Thymic epithelial tumours: ESMO Clinical Practice Guidelines for diagnosis, treatment and follow-up. Ann Oncol.

[17] Gentili F, Monteleone I, Mazzei FG, Luzzi L, Del Roscio D, Guerrini S, Volterrani L, Mazzei MA. Advancement in diagnostic imaging of thymic tumors. Cancers. 2021;13:3599. 10.3390/cancers13143599 PMC8303549 34298812

[18] Zhao Y, Chen H, Shi J, Fan L, Hu D, Zhao H (2015). The correlation of morphological features of chest computed tomographic scans with clinical characteristics of thymoma. Eur J Cardiothorac Surg.

[19] Yamazaki M, Oyanagi K, Umezu H, Yagi T, Ishikawa H, Yoshimura N, Aoyama H (2020). Quantitative 3D shape analysis of CT images of Thymoma: a comparison with histological types. AJR Am J Roentgenol.

[20] Qu YJ, Liu GB, Shi HS, Liao MY, Yang GF, Tian ZX (2013). Preoperative CT findings of thymoma are correlated with postoperative Masaoka clinical stage. Acad Radiol.

[21] Grivennikov SI, Greten FR, Karin M (2010). Immunity, inflammation, and cancer. Cell.

[22] Wu SD, Ma YS, Fang Y, Liu LL, Fu D, Shen XZ (2012). Role of the microenvironment in hepatocellular carcinoma development and progression. Cancer Treat Rev.

[23] Diakos CI, Charles KA, McMillan DC, Clarke SJ (2014). Cancer-related inflammation and treatment effectiveness. Lancet Oncol.

[24] Lee K, Hwang H, Nam KT (2014). Immune response and the tumor microenvironment: how they communicate to regulate gastric cancer. Gut Liver.

[25] Comen EA, Bowman RL, Kleppe M (2018). Underlying causes and therapeutic targeting of the inflammatory Tumor Microenvironment. Front Cell Dev Biol.

[26] Guo L, Wang Q, Chen K, Liu HP, Chen X (2021). Prognostic value of combination of inflammatory and tumor markers in Resectable Gastric Cancer. J Gastrointest Surg.

[27] Gu Y, Zheng F, Zhang Y, Qiao S (2022). Novel Nomogram based on inflammatory markers for the Preoperative Prediction of Microvascular Invasion in Solitary Primary Hepatocellular Carcinoma. Cancer Manag Res.

[28] Wang L, Wang C, Wang J, Huang X, Cheng Y (2017). A novel systemic immune-inflammation index predicts survival and quality of life of patients after curative resection for esophageal squamous cell carcinoma. J Cancer Res Clin Oncol.

[29] Feng XL, Lei XB, Dong WT, Yan LF, Xin YK, Li GF, Jing Y, Duan SJ, Zhang J, Hu YC, Li B, Zhao SS (2018). Incidence and clinical variable inter-relationships of thymic epithelial tumors in northwest China. J Thorac Dis.

[30] Choe J, Lee SM, Lim S, Choi SH, Kim N, Do KH, Seo JB (2017). Doubling time of thymic epithelial tumours on CT: correlation with histological subtype. Eur Radiol.

[31] Sadohara J, Fujimoto K, Müller NL, Kato S, Takamori S, Ohkuma K, Terasaki H, Hayabuchi N (2006). Thymic epithelial tumors: comparison of CT and MR imaging findings of low-risk thymomas, high-risk thymomas, and thymic carcinomas. Eur J Radiol.

[32] Tomiyama N, Johkoh T, Mihara N, Honda O, Kozuka T, Koyama M, Hamada S, Okumura M, Ohta M, Eimoto T, Miyagawa M, Müller NL (2002). Using the World Health Organization classification of thymic epithelial neoplasms to describe CT findings. AJR Am J Roentgenol.

[33] Pan CC, Chen WY, Chiang H (2001). Spindle cell and mixed spindle/lymphocytic thymomas: an integrated clinicopathologic and immunohistochemical study of 81 cases. Am J Surg Pathol.

[34] Hosseini S, Mofrad AME, Mokarian P, Nourigheimasi S, Azarhomayoun A, Khanzadeh S, Habibzadeh S, Ghaedi A (2022). Neutrophil to lymphocyte ratio in Epilepsy: a systematic review. Mediators Inflamm.

[35] Li L, Zhang H, Feng GL (2022). Neutrophil-to-lymphocyte ratio predicts in-hospital mortality in Intracerebral Hemorrhage. J Stroke Cerebrovasc Dis.

[36] Su M, Ouyang X, Song Y (2022). Neutrophil to lymphocyte ratio, platelet to lymphocyte ratio, and monocyte to lymphocyte ratio in depression: a meta-analysis. J Affect Disord.

[37] Xing Y, Tian Z, Jiang Y, Guan G, Niu Q, Sun X, Han R, Jing X (2022). A practical nomogram based on systemic inflammatory markers for predicting portal vein thrombosis in patients with liver cirrhosis. Ann Med.

[38] Zhu X, Zhou J, Zhu Y, Yan F, Han X, Tan Y, Li R (2022). Neutrophil/lymphocyte, platelet/lymphocyte and monocyte/lymphocyte ratios in schizophrenia. Australas Psychiatry.

[39] Ouyang H, Wang Z (2022). Predictive value of the systemic immune-inflammation index for cancer-specific survival of osteosarcoma in children. Front Public Health.

[40] Tong YS, Tan J, Zhou XL, Song YQ, Song YJ (2017). Systemic immune-inflammation index predicting chemoradiation resistance and poor outcome in patients with stage III non-small cell lung cancer. J Transl Med.

[41] Li P, Li H, Ding S, Zhou J, NLR (2022). PLR, LMR and MWR as diagnostic and prognostic markers for laryngeal carcinoma. Am J Transl Res.

[42] Wang L, Ruan M, Yan H, Lei B, Sun X, Chang C, Liu L, Xie W (2020). Pretreatment serum neutrophil-to-lymphocyte and monocyte-to-lymphocyte ratios: two tumor-related systemic inflammatory markers in patients with thymic epithelial tumors. Cytokine.

